# Mast Cell Involvement in Fibrosis in Chronic Graft-Versus-Host Disease

**DOI:** 10.3390/ijms22052385

**Published:** 2021-02-27

**Authors:** Ethan Strattan, Gerhard Carl Hildebrandt

**Affiliations:** Division of Hematology and Blood & Marrow Transplant, Markey Cancer Center, University of Kentucky, Lexington, KY 40536, USA; ethan.strattan@uky.edu

**Keywords:** mast cells, GVHD, fibrosis, transplant, autoimmune, pathogenesis

## Abstract

Allogeneic hematopoietic stem cell transplantation (HSCT) is most commonly a treatment for inborn defects of hematopoiesis or acute leukemias. Widespread use of HSCT, a potentially curative therapy, is hampered by onset of graft-versus-host disease (GVHD), classified as either acute or chronic GVHD. While the pathology of acute GVHD is better understood, factors driving GVHD at the cellular and molecular level are less clear. Mast cells are an arm of the immune system that are known for atopic disease. However, studies have demonstrated that they can play important roles in tissue homeostasis and wound healing, and mast cell dysregulation can lead to fibrotic disease. Interestingly, in chronic GVHD, aberrant wound healing mechanisms lead to pathological fibrosis, but the cellular etiology driving this is not well-understood, although some studies have implicated mast cells. Given this novel role, we here review the literature for studies of mast cell involvement in the context of chronic GVHD. While there are few publications on this topic, the papers excellently characterized a niche for mast cells in chronic GVHD. These findings may be extended to other fibrosing diseases in order to better target mast cells or their mediators for treatment of fibrotic disease.

## 1. Introduction

Chronic graft versus host disease (cGVHD) is a late complication that occurs in about 50 percent of patients that have undergone allogeneic hematopoietic stem cell transplant (allo-HSCT). In contrast to acute graft versus host disease (aGVHD), which is characterized by acute inflammation, massive cytokine production, and barrier dysfunction, cGVHD appears much more autoimmune in its mechanisms of pathogenesis. It is complex and poorly understood but is known to involve crosstalk and interactions between T-cells, B-cells, neutrophils, and tissue resident cells. One of the primary characteristics of cGVHD is inappropriate collagen deposition induced by an aberrant wound-healing mechanism [1]. Post conditioning, tissue-resident cells attempt to regulate the initial cytokine storm of aGVHD through production of anti-inflammatory cytokines and fibrogenetic factors to repair tissue damage. This production of anti-inflammatory mediators, while initially helpful in controlling alloreactive immune responses, leads to inappropriate collagen deposition and fibrotic disease.

cGVHD can affect any organ system, with key symptoms often related to increased collagen deposition. Histologically, these changes are often seen to be acellular, although this can change depending on the disease presentation. This fibrotic phenotype is an irreversible end-stage disease, and while symptom management has improved, overall incidence has not decreased [1]. For instance, lung involvement of cGVHD has been underestimated for decades [2]. Both restrictive and obstructive lung function changes are found, and bronchiolitis obliterans syndrome (BOS) is now understood as a classical form of cGVHD of the lung. It is one of the only pathognomonic symptoms of cGVHD and is associated with particularly poor prognosis due to these irreversible structural changes in the architecture of the lung [3].

In cGVHD, the most frequently involved organ is the skin, with symptoms often presenting as sclerosis alongside increased collagen deposition [4,5]. Scleroderma-like cGVHD observed in transplant survivors can result in skin thickening and stiffness at localized sites or can manifest over extended areas of the body which can lead to full loss of mobility and entire body encasement. Other cutaneous manifestations of cGVHD include lichen planus-like disease, morphea-like features, depigmentation, erythema, and ulceration [6,7]. In addition to the social stigma of widespread skin disease, these manifestations can have a significant negative impact on patient quality of life. Histologically, sclerotic dermal cGVHD presents as a thickening of the dermis due to massively increased collagen deposition. This collagen layer is relatively acellular, though fibroblasts are still present alongside scattered infiltrating cells [7]. Overall, the progression of fibrotic cGVHD is incompletely understood. While others have reviewed the field as it is currently known [1,8,9], there are still gaps in knowledge that need to be filled in order to better treat this progressive and debilitating disease.

## 2. Canonical Immune Involvement in GVHD

GVHD has traditionally been defined as a disease driven by alloreactive donor-derived CD4 and CD8 T-cells responding to non-self antigens presented by host-derived antigen-presenting cells (APCs) and target tissues [10]. While this is certainly true in aGVHD, in recent years, studies have shown a heavy level of B-cell involvement in cGVHD as well, particularly germinal center B-cells through their IL-21 mediated interactions with T-follicular helper cells [11]. While relative contributions are difficult to deconvolute, it is abundantly clear that adaptive immunity is a major driver of pathogenesis in both acute and chronic GVHD. Furthering this idea is evidence that IgE may be correlated with disease onset and severity [12,13]. IgE antibody is produced through a highly regulated, long-term, low dose antigenic response, requiring a very specific set of signals [14,15]. Therefore, IgE presence in this context predicates the possibility of mast cell involvement as well as overall dysregulation of an adaptive immune response.

The cytokine storm phenotype that can often occur in the early phases of aGVHD potentiates host-derived APCs to activate donor-derived T-cells. This occurs through presentation of self-antigens, which induces alloreactive donor T-cell activation and proliferation, followed by their trafficking to the tissues and causing many of the pathologies associated with GVHD.

Th1, Th2, and Th17 cells have all been shown to play a role in pathogenesis of GVHD. In aGVHD, proinflammatory Th1 cells are the primary T-cells involved, while in cGVHD, Th2 and Th17 cells have both been shown to play a role. This takes place especially through production of IL-17A, IL-4, and IL-13, which can promote fibrosis and production of alloreactive antibodies [16]. In aGVHD, a unique subset (non Th1, Th2, Th17) of granzyme A-producing Th cells has also been shown to further disease progression through a STAT3-dependent mechanism [17].

T follicular helper (Tfh) cells seem to be more directly involved in fibrotic manifestations of GVHD. ICOS+, IL-21-producing Tfh cells are known to contribute to skin fibrosis in other models of scleroderma [18], and this appears to be the case in cGVHD as well. Flynn et al. showed that Tfh cells produce IL-21 in cGVHD, which promotes germinal center formation and autoantibody production from GC B-cells, thereby increasing disease burden. Inhibition of this process through IL-21 blockade reversed this finding [11].

In contrast to other T-cells, Tregs are a cell type involved in tissue homeostasis and downregulation of inflammation. Almost universally in sites of GVHD-mediated tissue damage, there is a significant increase in the T-effector:Treg ratio. Homeostatic control of GVHD can be maintained in settings that promote Treg survival and proliferation or after exogenous addition of Tregs. Given the differences in the tissue environment between acute and chronic GVHD, it is perhaps surprising that Tregs seem to be critical for reduction of disease in both settings [19]. In particular, the mechanisms of disease onset in acute vs. chronic GVHD are often dissimilar; hence, a “regulatory” cell type implicated in pathogenesis of both diseases may be unexpected. However, it is important to recall that GVHD is primarily a disease of immune dysregulation (with both pathologic overactivation and excessive wound-healing leading to fibrosis), and therefore, Treg failure to dictate control over other elements of the immune system can lead to issues in both acute and chronic GVHD [20,21].

B-cells are also implicated in GVHD pathogenesis. For acute GVHD, the evidence of B-cell involvement is not as strong as for chronic GVHD; recipient B-cells are thought to be eventually eliminated after HSCT and donor B cells are not as significant of a proportion of the graft as T-cells. In combination with the evidence that GC B-cells may be drivers of pathogenesis in cGVHD, it seems that these are donor-derived B-cells that may not be numerous or mature enough to play a major role in early aGVHD. As a proof of concept, a drug targeting the protein BTK, is the only FDA-approved drug for chronic GVHD and has been shown to reduce GVHD and enhance graft-versus-leukemia (GVL) in murine models [22]. In cGVHD, BAFF signaling is found to be hugely important for B-cell pathogenesis, with long-term overexpression of BAFF levels alongside antigenic signaling driving B-cell proliferation and antibody production [23].

Of course, as GVHD is such a heterogenous disease, different aspects of adaptive immunity can be at play in local sites within the same patient. This makes biomarker studies particularly difficult. For instance, liver and skin fibrosis can both occur in cGVHD, but seem to do so by different mechanisms, with Tfh cells being more important for scleroderma, and Th17 cells being critical for development of liver fibrosis [24].

## 3. Historical Perspective on Mast Cells in Fibrotic Chronic GVHD

However, this current understanding of the pathogenesis of cGVHD has been insufficient to prevent or treat GVHD in some patients, many of whom can become refractory to long-term steroids or suffer other debilitating side effects [25]. A deeper knowledge of the pathogenic factors at play in cGVHD is necessary to change patient outcomes. One of the first to pinpoint mast cells in fibrotic cGVHD was Henry Claman, a seminal immunologist whose lab was responsible for the vast majority of early work on this topic. In 1985, he described the disappearance of toluidine blue-stained mast cells in formalin-fixed paraffin-embedded skin from Balb/C mice after irradiation and injection of B10.D2 marrow [26]. This is now established as a model of sclerodermatous cGVHD but had not been extensively explored at the time. Indeed, Claman’s use of this model was predicated on its utility as a model of progressive scleroderma, a phenomenon that was only poorly explicable at the time.

However, there are several confounding factors at play that may explain the lack of staining. To begin with, it has previously been shown that activation status due to malignancy can cause changes in toluidine blue-induced metachromasia in mast cells in a pH-dependent manner [27,28]. In a previous study using a range of pH values, proliferating malignant mast cells stained much more effectively at pH > 3.5, while in Claman’s study the pH of the toluidine blue solution was 3.2. In the discussion, the authors mention that ultrastructural staining of mast cells in the skin showed degranulation due to mast cell activation. While toluidine blue staining would be lessened due to mast cell degranulation, it is possible that staining with toluidine blue at a higher pH would have better distinguished these cells. Other methods, such as staining with an FcεR1α antibody, may also have yielded clearer results, but these reagents had yet to be developed. Therefore, ultrastructural analysis of mast cell morphology and cell contents in the skin was the gold standard assay at the time.

While the mast cell disappearance in this study was not unexpected given the high dose of irradiation (9Gy) and the paradigm of host cell elimination during conditioning, Claman was nonetheless unsure of whether his staining methods were accurately capturing the mast cell phenotype in this model. He hypothesized that mast cells may not be eliminated, but rather degranulated, and therefore difficult to stain and identify with toluidine blue. In 1986, he followed up on this hypothesis, demonstrating that mast cells did in fact disappear from skin staining by toluidine blue after onset of the graft-versus-host reaction, but they were not eliminated. However, when the skin was examined by ultrastructural analysis via electron microscopy, mast cells were clearly still present, albeit degranulated. Claman referred to these as “phantom mast cells” and demonstrated that they showed an activated phenotype and were slowly rebuilding their granule contents [29].

Looking back at these studies from the span of several decades, it is clear that there was more to this story than Claman initially suspected. Given many of the characteristics seen, we suspected that these studies may hint at some GVHD biology that had been understudied. The degranulation of mast cells early in the onset of GVHD symptoms allows for a convenient trigger for the initial inflammatory cascade, which can then be further propagated in the context of the alloreactive feedback loop known to exist in aGVHD. Further, the fact that skin mast cells degranulate and rebuild their granule contents rather than merely disappearing allows for the possibility that recipient-derived mast cells have survived conditioning, although these studies were too nonspecific to rigorously answer that question. Additionally, if these mast cells are resistant to irradiation, is it possible that they may also survive other forms of conditioning, such as chemotherapy-based regimens? Lastly, given this activated mast cell phenotype alongside the clinical evidence of scleroderma, is it possible that mast cells are important in the progression of this disease?

However, for several years after this work, the study of mast cells in GVHD pathogenesis languished. Interest was revived by Francesca Levi-Schaffer while working with Henry Claman. While Claman noted the activated mast cell phenotype, Levi-Schaffer dug further into the mechanism. Beginning a theme that would be repeated throughout her career, Dr. Levi-Schaffer focused on mast cell, eosinophil, and fibroblast interactions, a feedback loop involving a grouping of cells her lab has dubbed the “allergic effector unit” (AEU) [30]. This process begins with mast cell activation and leads to eosinophil recruitment and fibroblast activation, ultimately ending in pathogenic fibrosis throughout the affected organ system, an outcome that looks strikingly similar to the end-state of fibrotic cGVHD.

In the early 1990′s, Dr. Levi-Schaffer began to examine the dynamics of mast cell activation in cGVHD. Using murine mast cells in vitro, she established a system wherein she cocultured mast cells with splenic supernatants from mice that had established cGVHD in addition to supernatants from healthy mice. When incubated with cGVHD supernatants only, these coculture systems induced mast cell activation and degranulation, implying that something in the splenic environment during cGVHD is strongly capable of activating mast cells [31]. Expanding on this, when she isolated fibroblasts from mice with cGVHD and cocultured them with mast cells, she saw mast cell proliferation and activation [32]. Given that we now understand the AEU to begin with mast cells and end with fibroblast activation, this hinted at a possible feedback loop of mast cell and fibroblast activation in cGVHD.

However, while the cGVHD setting was unique in these studies, they were far from the first to study mast cell and fibroblast interactions. Since both cell types are so often colocalized, early studies actually hypothesized that fibroblast-derived factors induced mast cell differentiation [33,34]. It is also known that mast cells produce substantial amounts of basic fibroblast growth factor through degranulation [35,36,37], a key mediator in induction of fibroblast proliferation and activation. Therefore, we believe that mast cells, when activated during the graft-versus-host reaction, induce fibroblast proliferation through secretion of activating factors.

We examined this further in our studies [38], finding that supernatants from activated mast cells did indeed induce fibroblast proliferation when compared to supernatants from quiescent mast cells. This lends credence to the idea of a mast cell/fibroblast feedback loop in cGVHD. These historical studies from Claman and Levi-Schaffer established a basis for future experiments, yet as striking as these studies were, they still had not examined the role of mast cells in vivo in cGVHD.

## 4. Mast Cells as Immunoregulatory Cells in Acute GVHD

While mast cells are thought to be involved in fibrotic disease under certain conditions, there had been few notable in vivo studies on their involvement in GVHD until 2013, when Leveson-Gower and Robert Negrin published on an immunomodulatory role for mast cells during the onset of aGVHD. They used a fully major histocompatibility complex (MHC)-mismatched model of GVHD and showed that the absence of mast cells led to significant increases in GVHD severity compared to WT mice [39]. However, this phenomenon was at least partially dependent on IL-10 production by mast cells.

At first glance, this immunomodulatory role seems as if it does not fit with the idea of pathogenic and profibrotic mast cells, although it is in fact of great interest. Mast cells are a cell type heavily involved in allergy, which is notoriously skewed towards Type 2 or Th2 immunity [40]. Therefore, mast cell production of IL-10, a cytokine that is often found to be implicated in Th2 responses, fits with their previously understood role. Additionally, while aGVHD is known for its inflammatory (Type 1) nature, cGVHD tends to be much more dominated by Type 2 responses. Therefore, an immunomodulatory mast cell, while initially helpful in the burst of inflammation accompanying aGVHD onset, may continue to act in a Type 2 manner; aberrant and prolonged Type 2 responses are known to result in pathogenic fibrosis similar to that seen in cGVHD [41].

## 5. Mouse Models of Fibrotic Chronic GVHD

In humans, GVHD can manifest in many different organ systems and display an incredibly wide range of clinical symptomology. For decades, researchers have used murine models of bone marrow transplant as a surrogate for human disease. These models are performed by transplanting bone marrow and/or splenocytes harvested from a donor mouse into a recipient mouse that has undergone conditioning and are reviewed excellently by Schroeder et al. [42]. This conditioning, as in patients undergoing HSCT, is most often based on chemotherapy or total body irradiation.

Used as a negative control that does not show the disease phenotype, syngeneic transplants are readily achievable in murine models by using genetically identical mice of the same strain. Allogeneic transplants are often used to model graft-versus-host disease, the severity, penetrance, and organ-specific manifestations of which depend on the degree of MHC-matching between the donor and recipient, the T cell dose, and the conditioning intensity. Murine allogeneic transplants, while they do not individually capture the entire clinical spectrum seen in human disease, allow for a more focused analysis of specific aspects of GVHD. As with GVHD itself, these models are broadly subtyped into those that induce acute or chronic disease, and GVHD phenotype is to some extent strain dependent.

Mouse models of cGVHD attempt to recapitulate the smoldering inflammation, thymic dysfunction leading to dysfunctional Treg cells, and pathogenic fibrosis often seen in patients [43]. Sclerodermatous disease is most common in the skin, and two minor histocompatibility antigen (miHag)-mismatched mouse models of transplant (total body irradiation (TBI) conditioning followed by B10.D2 → BALB/C [44] or LP/J → C57BL/6 [45]) display extensive scleroderma and minimal mortality, with fibrosis and dermal thickening evident from day 30 onward. Autoantibody deposition leading to lupus-like symptoms are also characteristic of cGVHD and can be modeled with the DBA2 → B6D2F1 model, which results in splenomegaly due to B-cell expansion [42].

## 6. In Vivo Studies of Fibrotic Mast Cells in Chronic GVHD

Our lab desired to study these phenomena in vivo using mast cell-deficient mice in a murine model of cGVHD [38]. The most commonly used strain of mast cell-deficient mice is derived from the C57BL/6 mouse [46]. Therefore, we used the LP/J → C57BL/6 model of cGVHD, a model characterized by progressive and systemic dermal sclerosis [45]; we have already seen evidence that mast cells may be involved in such processes. Mice were conditioned with 8.5Gy TBI followed by engraftment of allogeneic donor cells. Wild-type (WT) recipients (allo-WT) displayed significantly increased levels of cGVHD symptomology compared to mast cell (MC)-deficient recipients (allo-MCd). A classical feature of dermal cGVHD is skin thickening, especially due to significant collagen deposition in the dermal layer. We therefore examined the dermis of these mice by Masson’s trichrome staining; a thin, diffuse, and well-ordered layer of collagen was visible in the allo-MCd animals, while the allo-WT animals had a much thicker and denser layer of collagen along with a significant increase in pro-fibrotic gene expression. It was also shown that the mast cells involved in these processes were likely recipient-derived cells that had survived conditioning and the post-transplant environment. We examined this topic in vitro, finding that mast cells displayed substantial resistance to both ionizing radiation and busulfan/cyclophosphamide-based conditioning regimens, which could be a mechanism of their survival in this model [38].

Of particular interest was the mast cell-dependent dysregulation of chemokine signaling and immune infiltration found in the skin of allo-WT animals. Genes involved in chemokine signaling along with protein levels of CCL2, CCL3, CCL4, and CCL5 were broadly increased in allo-WT vs. allo-MCd recipients. We showed that mast cells could produce many of these same chemokines upon activation and that this was modulated by treatment with ibrutinib and ruxolitinib, drugs used clinically to treat steroid-refractory cGVHD. Given the clinical efficacy of these drugs in treatment of cGVHD and other fibrosing diseases, it is possible that modulation of mast cell function and chemokine production may be part of their mechanism of action. These drugs should be further investigated for their effect on fibrotic disease and mast cell function in other contexts. This study also examined skin biopsies from patients with dermal cGVHD. Patients with cGVHD had more mast cells in the skin than healthy controls. Of course, while any retrospective clinical study is correlative in nature, this does lend credence to mast cells being involved in both human and murine cGVHD. This study is the first in vivo evidence of fibrotic mast cells in a cGVHD model. It is also, to our knowledge, the only study broadly examining clinical outcome and correlations with cellular and molecular phenotype in this context. However, the field is very open for follow-up studies, with mechanisms and molecular targets needing significant characterization. Additional mouse models of mast cell deficiency and examination of mast cells in other models of GVHD will be crucial in verifying the role of mast cells in vivo in fibrotic cGVHD.

## 7. Future Directions for the Study of Mast Cells in Chronic GVHD

Expanding on these studies will be important, but there is now a significant body of literature demonstrating that mast cells can be important instigators of fibrotic disease. However, additional data are needed in diverse mouse models to further define their role. Most current studies have been performed in the B6.Cg-KitW-sh/HNihrJaeBsmGlliJ mast cell-deficient mice. This strain is well characterized and has been used to model the role of mast cells across many diseases. However, strictly isolating the role of mast cells using this model can be problematic due to the nature of the genetic changes in this model that allow for mast cell-deficiency. Most murine models of mast cell-deficiency are based around disruptions of the *Kit* gene, but this particular model has a genetic inversion covering several dozen genes, including some that are involved in immune function [46,47]. This can therefore lead to a false signal if one of the other genes is critical for disease processes independent of mast cells [48]. This raises another point; while there now exists a significant amount of data on the effects of mast cell-deficiency in cGVHD, much of it could be attributed to secondary effects. This could be, as mentioned, due to genetic factors involved in the *Kit* inversion. Another possibility is that while mast cells seem to be involved in creating a pro-inflammatory environment early on in the disease pathogenesis, they may be less critical once an inflammatory infiltrate is established. Therefore, once present in the tissues, more canonical GVHD effector cells such as neutrophils or T-cells may be capable of driving the disease even in the absence of mast cells. In addition to other models of mast cell deficiency, studies using mice from diverse genetic backgrounds will further inform our understanding of this topic. Common laboratory mice strains are often skewed towards Type 1 or Type 2 immunity, with the commonly-used C57BL/6 mice more Th1 dominant than a canonical Th2-skewed mouse such as Balb/C [49,50,51,52]. This phenomenon seems to affect mast cells as well as other immune cells, although there is currently no literature expounding on the effect that this may play in studies of fibrotic mast cells in GVHD. Therefore, analysis of mast cells and their role in fibrosis across multiple genetic backgrounds is critical to better understand the biology behind this phenomenon and to more effectively treat diverse manifestations of fibrotic disease.

In addition, there is a severe lack of specific inhibitors of mast cell function. Where inhibitors exist, they are either nonspecific or poorly characterized outside of their effect on mast cell function. For instance, cromolyn sodium, a “mast cell stabilizer,” is significantly more inhibitory in rats than in mice, despite the overall similarity of mast cell levels between species [53]. The same study also showed that cromolyn inhibited LPS responses in genetically mast cell-deficient mice, implying that it exerts effects outside of the mast cell lineage. It has been known for decades that cromolyn also inhibits macrophages function [54], and it is capable of binding to a variety of “off-target” proteins, including G-protein coupled receptors and heat-shock proteins [55,56]. Studying the effects of other drugs that inhibit mast cell responses is critical. Ibrutinib is FDA-approved for treatment of steroid-refractory cGVHD [57], and ruxolitinib has recently finished its Phase 3 trial for the same indication, meeting both primary and secondary endpoints [58]. These drugs are potentially game-changing in the treatment and management of cGVHD. Our data demonstrate that mast cells produce many of the same chemokines seen to be significantly upregulated after allogeneic transplant, and these drugs allow for modulation of this mast cell activity. However, while they inhibit many aspects of mast cell activation, they are similarly nonspecific. Mast cells, alongside many other immune cells, use the ibrutinib and ruxolitinib target proteins (BTK/ITK and Jak1/Jak2, respectively) as key mediators in cytokine signal transduction and FcεR1α-mediated activation. Future work is needed to characterize whether the clinical effectiveness of these drugs is, at least in part, due to their inhibition of mast cell responses.

Another area of interest for future studies would be to determine the mechanism of activation of mast cells in the post-transplant environment. It is currently unknown as to the method or methods by which mast cells are activated in GVHD. While serological studies, highlighted in more detail above, indicate the possible presence of an IgE-mediated response, it is more likely a combination of activating elements that lead to the phenotype observed in GVHD. IL-33, for instance, is a potent activator of mast cells and is produced during tissue damage such as that seen in GVHD [59]. TGF-beta, a commonly implicated profibrotic cytokine even in GVHD [60], also exerts its effects directly on mast cells. Interestingly, it induces downregulation of FcεR1α [61] and suppresses IL-33 signaling in MCs [62]. When our group examined TGF-beta in our in vivo model of murine cGVHD, we saw no difference in TGF-beta protein levels in the skin. Taken together, this leads us to believe that TGF-beta may not be directly responsible for MC activation in this system, although more data is needed. Other methods of mast cell activation, such as MRGPRX2-stimulation [63,64,65], also need to be considered. Therefore, a broad and unbiased approach is needed to identify the signaling pathways involved in mast cell activation and the role of mast cells in fibrosis in these systems.

While a superficial analysis of MC degranulation can look similar between stimuli, transcriptional or kinetic analysis reveal fundamental differences in MC function after activation [66,67,68]. Using the signals defined in this previous tranche of literature, analysis of MC populations in GVHD target organs could therefore indicate the methods by which mast cells are activated in the tissues. Single cell RNA-sequencing may be an important tool in this process, especially when coupled with fibrotic disease models that show mast cell dependence. Broader approaches in similar murine models would also be helpful; deletion of genes involved in mast cell activation would allow for quick screening of potential pathways. The increasing availability of both conditional and constitutive mast cell-deficient mice across a variety of genetic backgrounds makes these studies more feasible than ever before [69,70,71,72,73].

Ultimately, the studies outlined here are only a beginning. While we have seen that many of the features of murine sclerodermatous chronic GVHD are dependent on mast cells, there are still many questions in regard to their mechanism of activation, their survival after allogeneic transplant, and their normal physiological roles. While the mast cell, particularly in the context of allergy, is viewed as relatively one-dimensional, these studies put the lie to that paradigm.

## Abbreviations

HSCT	Hematopoietic stem cell transplant
GVHD	Graft-versus-host disease
aGVHD	Acute graft-versus-host disease
cGVHD	Chronic graft-versus-host disease
MC	Mast cell
allo-HSCT	Allogeneic hematopoietic stem cell transplant
BOS	Bronchiolitis obliterans syndrome
Gy	Gray (unit of radiation)
AEU	Allergic-effector unit
MHC	Major histocompatibility complex
miHag	Minor histocompatibility antigen
allo-WT	Allogeneic transplant—Wild type recipients
allo-MCd	Allogeneic transplant—Mast cell-deficient recipients
